# Ontogenetic Plasticity in Shoaling Behavior in a Forage Fish under Warming

**DOI:** 10.1093/icb/icad043

**Published:** 2023-05-27

**Authors:** Fidji Berio, Camille Morerod, Xuewei Qi, Valentina Di Santo

**Affiliations:** Department of Zoology, Stockholm University, Svante Arrhenius väg 18B, 114 18, Stockholm, Sweden; Department of Zoology, Stockholm University, Svante Arrhenius väg 18B, 114 18, Stockholm, Sweden; Department of Zoology, Stockholm University, Svante Arrhenius väg 18B, 114 18, Stockholm, Sweden; Department of Zoology, Stockholm University, Svante Arrhenius väg 18B, 114 18, Stockholm, Sweden

## Abstract

Shoaling behavior is known to increase survival rates during attacks from predators, minimize foraging time, favor mating, and potentially increase locomotor efficiency. The onset of shoaling typically occurs during the larval phase, but it is unclear how it may improve across ontogenetic stages in forage fishes. Warming is known to increase metabolic rates during locomotion in solitary fish, and shoaling species may adjust their collective behavior to offset the elevated costs of swimming at higher temperatures. In this study, we quantified the effects of warming on shoaling performance across the ontogeny of a small forage fish, zebrafish (*Danio rerio*) at different speeds. Shoals of larval, juvenile, and adult zebrafish were acclimated at two temperatures (28°C and 32°C), and metabolic rates were quantified prior to and following nonexhaustive exercise at high speed. Shoals of five individuals were filmed in a flow tank to analyze the kinematics of collective movement. We found that zebrafish improve shoaling swimming performance from larvae to juveniles to adults. In particular, shoals become more cohesive, and both tail beat frequency (TBF) and head-to-tail amplitude decrease with ontogeny. Early life stages have higher thermal sensitivity in metabolic rates and TBF especially at high speeds, when compared to adults. Our study shows that shoaling behavior and thermal sensitivity improve as zebrafish shift from larval to juvenile to adult stages.

## Introduction

Shoaling refers to a behavior in which fish of the same species aggregate and swim together. Shoaling behavior is one of the most remarkable collective motions observed in the majority of fish species at least at some point in their life. This social behavior favors the detection of food and mates and increases the survival rates of individuals when facing predators (Pitcher 2001; Hemelrijk et al. 2015). In fact, fish in a shoal can confuse predators by splitting and swimming in different directions during an attack (Zamon 2001). Even though it is broadly accepted that fishes form shoals for social reasons, some may swim in polarized and synchronized formations to lower the costs of locomotion, and these aggregations are known as schools (Pitcher 1986; Delcourt and Poncin 2012; Miller and Gerlai 2012). Schooling fishes select geometrically precise positions to take advantage of vortices shed by other individuals swimming anterior to them in the aggregation (Miller and Gerlai 2007; Di Santo 2022). However, there are no set criteria to classify a fish as either a “schooler” or “shoaler”. Instead, it is recognized that there is a continuum in swimming organization within fish aggregations, with precise positioning and short distances between individuals, typical of schooling, being considered the most energetically efficient collective behavior (Weihs 1973; Ashraf et al. 2017; Saadat et al. 2021).

Shoaling behavior is not readily exhibited in fishes after hatching but arises gradually over ontogeny (Ioannou and Laskowski 2023). This behavior is generally initiated during the larval stage when the fish are about 10–12 mm in length, or post-flexion (Shaw 1961; Miller and Gerlai 2007; Buske and Gerlai 2011). Assessing the development of shoaling in larvae is, therefore, crucial to get insights into the mechanisms driving this aggregative behavior. When investigating the onset of shoaling behavior, particular attention has been placed to quantifying polarization, that is, fish swimming in the same direction, and inter-individual distance. In the case of schooling, inter-individual distance provides a measure of the interactions in a formation (Weihs 1973, 1975; Katz et al. 2011). As larvae grow in size, inter-individual distance decreases and larvae assume a parallel position. In pelagic marine fishes like chub mackerels (*Scomber japonicus*), Pacific jack mackerels (*Trachurus symmetricus*), Californian anchovies (*Engraulis mordax*), jack silversides (*Atherinopsis californiensis*), and topsmelt silversides (*Atherinops affinis*), young stages (larval and juvenile) form less compact shoals and show a wider dissimilarity in body angle to the direction of swimming than adults, suggesting that schooling behavior may develop over ontogeny (van Olst and Hunter 1970).

Fishes respond to social signals and environmental cues as they move collectively; therefore, it is likely that stressors such as warming may significantly affect dynamics within shoaling across life stages. The effect of ocean warming has been investigated extensively on many processes and ecological aspects in fishes (Angilletta 2009; Somero 2010; Eliason et al. 2011; Hein and Keirsted 2012). Temperature is considered the “abiotic master factor” as it imparts strong direct and masked effects on virtually every physiological process in fishes (Fry 1947, 1967; Brett 1971), and many studies have quantified the relationship between physiological traits and temperature (Johnson and Bennett 1995; Bennett and Beitinger 1997; Di Santo and Lobel 2017; Andreassen et al. 2022). Small- and large-scale migrations of schools of pelagic fishes are influenced by changes in sea surface thermal gradients (Humston et al. 2000) and fishes are known to select temperatures to enhance physiological processes (Sims et al. 2006; Di Santo and Bennett 2011; Papastamatiou et al. 2015). Moreover, the collective behavior and distribution of forage fishes are known to be strongly affected by daily and seasonal changes in temperature (Abookire et al. 2000; Gobler et al. 2018; Kuruvilla et al. 2022). For instance, warming is known to reduce the inter-individual distance in forage fishes (Bartolini et al. 2015; Colchen et al. 2017); however, the response is species-specific and likely to depend on life stage, local adaptation, and acclimation history (Di Santo 2016). The loosening of a formation, as evident from a greater inter-individual distance, may cause a reduction in locomotion efficiency for the school because individuals might be less likely to take advantage of vortices shed by other fish (Saadat et al. 2021; Di Santo 2022; Lauer et al. 2022). Furthermore, warming can negatively impact cognitive functions affecting lateralization (the preference for right and left turns) and cohesiveness (Colchen et al. 2017; Mitchell et al. 2022). However, it is still unclear if warming affects shoaling behavior differently across the ontogeny of fishes. Therefore, deciphering the vulnerability of shoaling behavior to increases in temperature across life stages is key to identifying possible bottlenecks for the survival of most fish species in the context of global warming.

Zebrafish *Danio rerio* is a small freshwater forage species widely used to understand the development of collective behavior and the regulation of physiological processes in vertebrates (Briggs 2002; Miller and Gerlai 2007; Buske and Gerlai 2011; Shelton et al. 2020). Shoaling behavior in zebrafish develops during the first four weeks post-fertilization at the optimal temperature of 28°C (Buske and Gerlai 2011). Larvae begin to position themselves next and parallel to each other around 9 days post fertilization (dpf) and are already shoaling by 14 dpf (Hinz and de Polavieja 2017). This is important as, in the wild, zebrafish may encounter high flow conditions that impose energetic costs that this fish may try to reduce (Flierl et al. 1999). Additionally, warming is likely to increase metabolic costs in zebrafish, which could be partially offset by efficient locomotor behavior. However, to date, we do not understand the effect of warming on shoaling behavior kinematics across the ontogenetic stages of zebrafish. To address this question, we quantified ontogenetic shifts in shoaling behavior of zebrafish acclimated to two different temperatures (28°C, or control, and 32°C). We measured resting and post-swimming metabolic rates for each shoal to quantify the effect of warming on recovery metabolic rates after nonexhaustive swimming at high speed. We also analyzed major swimming kinematics in larvae (about 20 dpf), juveniles (about 1-month-old), and adults (over 1-year-old) at different speeds to determine the effects of warming and life stage on shoaling performance.

## Materials and methods

### Animals and temperature acclimation

*Danio rerio* larvae (4 dpf), juveniles (2-weeks-old), and adults (over 1-year-old) were obtained from Karolinska Institute and IMAZO (Sweden) and were held in an aquarium room kept at about 24°C, under the Animal care protocol number 11924-2020 approved by the Swedish Board of Agriculture. Fish across the life stages were divided into two temperature treatments, 28°C (control) and 32°C (± 0.5°C) (*n* = 4–5 replicate shoals, per stage at each temperature), and maintained in tanks as groups (Table 1).

**Table 1:** Shoal identification (ID) for larvae (L), juveniles (J), and adults (A).

Shoal ID	Temp.	Larvae	Juveniles	Adults
(L,J,A)	(°C)	(*n* = 5 fish)	(*n* = 5 fish)	(*n* = 5 fish)
1	32	32.0 ± 0.7	32.2 ± 0.4	31.9 ± 0.5
2	28	28.1 ± 0.2	28.1 ± 0.1	28.0 ± 0.2
3	32	32.1 ± 0.2	32.1 ± 0.2	32.2 ± 0.2
4	28	28.0 ± 0.2	27.9 ± 0.4	28.1 ± 0.3
5	32	32.0 ± 0.6	31.9 ± 0.2	31.9 ± 0.4
6	28	28.1 ± 0.1	28.1 ± 0.1	28.1 ± 0.1
7	32	32.0 ± 0.3	32.2 ± 0.1	31.8 ± 0.3
8	28	28.1 ± 0.2	28.1 ± 0.0	28.1 ± 0.2
9	32	32.1 ± 0.2	–	32.2 ± 0.2
10	28	28.0 ± 0.2	27.9 ± 0.4	28.0 ± 0.2

Larval (L), juvenile (J), and adult (A) shoals are composed of different individuals (*n* = 5 fish per shoal). Target acclimation temperature treatments (Temp. °C), as well as recorded values (means ± SD computed from daily measurements) for each shoal, are given.

The temperature in each tank was maintained with submersible heaters (Eheim thermocontrol 50), monitored, and recorded daily, across the whole acclimation time (Table 1). Fish were acclimated to their treatment temperatures for at least two weeks before the swimming experiments were performed. Larvae were maintained on a diet of enriched rotifers for the first ten days and were further fed on zebrafish pellets (<100 μm). Juveniles were fed a diet of pellets and adults were fed flakes (Tetra). Each tank was aerated and filtered independently, and water quality in each tank was maintained so that no ammonia or nitrites were detected. Water changes were performed weekly in each tank.

### Oxygen consumption rates

**Fig. 1:**
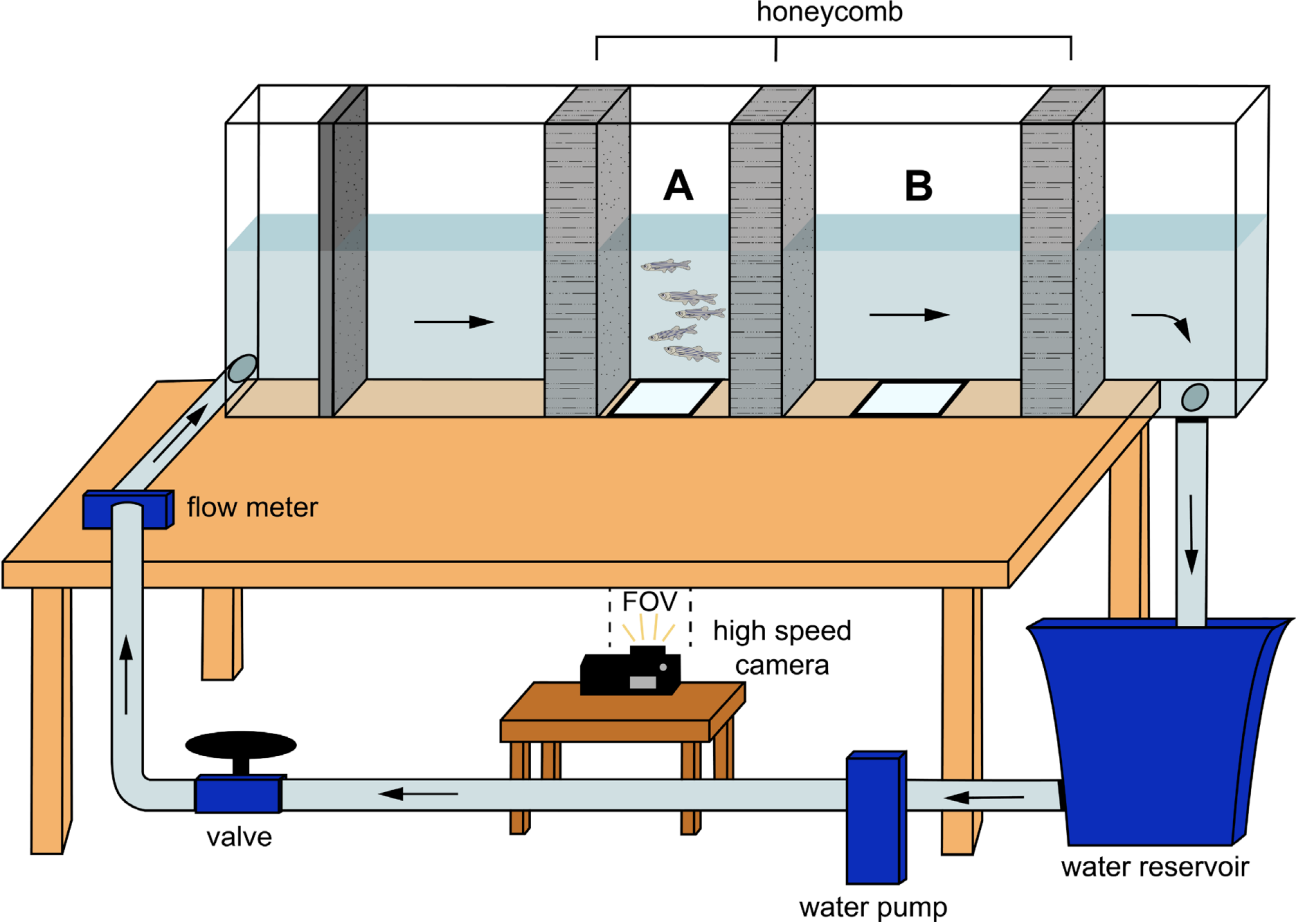
Flowtank set up used to investigate shoaling behavior of *Danio rerio* across different ontogenetic stages. (A) working section for larvae and juveniles, and (B) working section for adults. FOV, field of view.

Three different-sized respirometer chambers were used to quantify oxygen consumption rates: 7 mL (larvae), 26 mL (juveniles), and 615 mL (adults). Respirometers were placed in a water bath maintained at the appropriate temperature (28°C or 32°C). Oxygen concentration (*MO*_2_, *mgO*_2_*l*^−1^) over time and temperature were measured in the respirometer chamber every 60 s using a Pyroscience oxygen meter. Zebrafish were fasted for at least 24 h prior to metabolic measurements to ensure measurements were taken during postabsorptive state and then placed in the respirometer for 1 h. To reduce disturbance, a sheet of dark plastic was placed on the respirometer chambers. Our goal was to measure the metabolic rates of zebrafish prior to and during recovery from nonexhaustive exercise to understand the effect of temperature on recovery from swimming at different ontogenetic stages. Therefore, our measurements did not aim at quantifying minimum resting but rather resting routine metabolic rates. As a consequence, activity in the respirometers was not closely monitored, even though zebrafish under dark conditions tend to stay quiescent (Burgess and Granato 2007; Chiu and Prober 2013). Additionally, our goal was to measure the metabolic rate of the shoal rather than individual fish in the group. Therefore, five individuals per tank were considered the “unit” and placed in the respirometer together. Zebrafish routine metabolic rates (*mgO*_2_*l*^−1^*h*^−1^, *n* = 5 fish per shoal) were measured for 1 h prior to exercise. The fish were then placed in an open flow tank (Loligo Systems) (Fig. 1; working section: 8.5 × 20 cm for juveniles and larvae and 9 × 5 cm for adults) and allowed to acclimate for at least 15 min prior to the swimming exercise. Each shoal swam at a constant speed of 7 body lengths (BL) per second for 15 (larvae and juveniles) and 30 min (adults). After nonexhaustive exercise the shoal was returned to the respirometer chamber and recovery oxygen consumption rates were measured for at least 2  h.

**Fig. 2:**
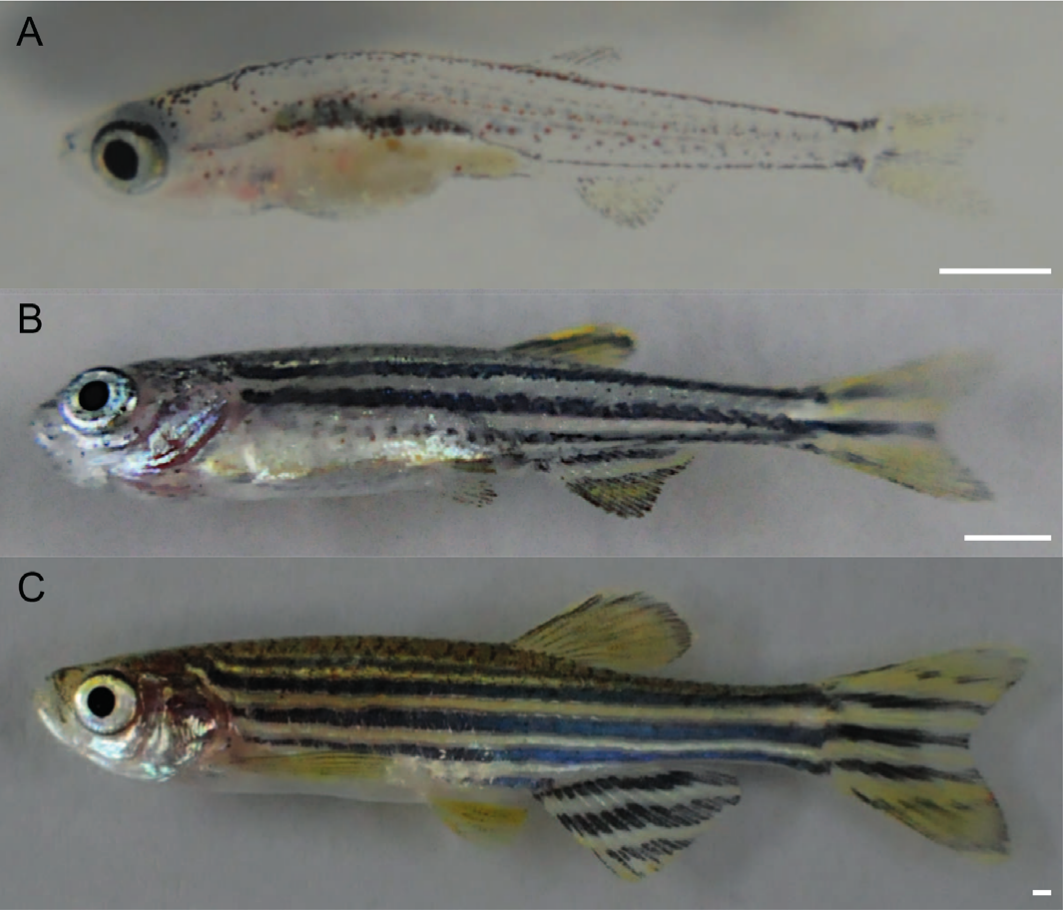
Ontogenetic shifts of shoaling were investigated in three stages in *Danio rerio*. (A) 23 days post fertilization larva; (B) 1 month juvenile; and (C) adult. Scale bars are 1 mm.

After the completion of oxygen consumption rates, larval, juvenile, and adult zebrafish were photographed and measured (Fig. 2).

All the *MO*_2_ values are given as *mgO*_2_*l*^−1^*h*^−1^. Mass measurements were not taken for larvae and juveniles because they would require sacrificing the fish. Instead, we present mean non-mass-adjusted oxygen consumption rates per individual per shoal. Factorial *MO*_2_, i.e. the ratio between *MO*_2_ at max peak during recovery and resting routine *MO*_2_, was calculated to compare across life stages and temperature treatments. Indeed, factorial rather than mass-adjusted metabolic rates are most likely to reveal the effect of temperature and stage on oxygen consumption before and during recovery from exercise because the metabolic requirements and muscle composition across the life stages of zebrafish vary (Killen et al. 2006).

Temperature quotients (*Q*_10_) were further estimated for metabolic rates to quantify thermal sensitivity. For all stages, *Q*_10_ values for metabolic rates at rest and at the peak during recovery from exercise were estimated between high and low temperatures (32°C and 28°C). Temperature quotients were calculated using mean rates per stage and temperature. The temperature quotient was determined using the following equation (Schmidt-Nielsen 1997): $Q_{10} = (K_{2} K_{1}^{-1}) ^{(10/ (T_{2}-T_{1}))}$, where *K*_1_ is the mean rate at 28°C, *K*_2_ is the mean rate at 32°C, *T*_1_ is 28°C, and *T*_2_ is 32°C.

### Shoal swimming kinematics

Zebrafish shoals from each stage and temperature treatment were tested at different speeds (Fig. 1): 1.5, 2.6, 3, 4, and 5 BL s^−1^ for larvae, 1.5, 2, 3, 4, and 5 BL s^−1^ for juveniles, and 1, 1.5, 2, 3, 4, and 5 BL s^−1^ for adults. The order of the speeds was randomized to avoid carry-over effects of swimming repeatedly and the recordings at different speeds were run sequentially for each school. At each speed, fish swam for about 1 min or less and 1–2 independent sequences were recorded at 1000 fps using a high-speed camera (Chronos 1.2, Krontech) placed ventrally. Behavioral characteristics of shoals such as mean separation distance (or the mean inter-individual distance for each fish in the shoal, in BL) and number of events where fish switched position were quantified (as *no*. s^−1^). Images from the video sequences were calibrated using ImageJ (Schneider et al. 2012) by direct linear transformation. Midlines (*n* = 12–13) were digitized for each individual in the shoal (Di Santo et al. 2021) using CurveMapper (code available in Goerig et al. 2021) in MATLAB (MathWorks, Natick, MA, USA). Midlines were composed of 200 points along the mid-body and covered one complete caudal fin cycle. Kinematics parameters from each individual in the shoal were averaged and one value per shoal, per flow speed was used in the analysis. Values taken from 1 or 2 independent video sequences were averaged for the analysis. Kinematics variables were extracted as described in Di Santo et al. (2021). Briefly, tail beat frequency (TBF, in Hz) was measured by dividing the frame rate by the total number of frames across one fin beat cycle, and head and tail amplitudes (as proportion of BL) were measured as the maximum lateral oscillation of the first and last five points in the midline (head and tail, respectively). Wave speed (in BL s^−1^) and wavelength (in BL) were obtained for each tail beat by analyzing the local curvatures of the body. Maximum curvature was quantified by iteratively using three points along the body at 5% BL distance from each other (Di Santo et al. 2021). Curvature is the inverse of the radius that describes the curve along these three points (*κ* = r^−1^).

### Data analysis

Mean values for kinematic and metabolic variables for each shoal were computed and compared using ANOVAs and permutation tests. Resting routine and peak metabolic rates were compared between the temperature treatments, within each stage using a One-Way ANOVA. Comparisons across stages were not possible because the metabolic rates were not mass-adjusted. Factorial metabolic rates were instead used to compare across stages and temperature using a Two-Way ANOVA. All behavioral and kinematic variables were tested across stages and warming within each speed using permutation tests. We report F statistics and *P*-values for the whole model, followed by the *P*-values of interaction and single factors effects. Each shoal was tested at different speeds. The speeds were not independent and equally replicated across shoals, we chose to test differences in locomotor behavior and kinematics within each speed. The statistical analyses are performed using R software (v4.2.1) with the lmPerm package (v2.1.0) (Wheeler et al. 2016).

## Results

### Animals and temperature treatments

Zebrafish were massed (g, adults) and measured (mm, all stages) after the acclimation period to the two temperature treatments (Table 2). The mass of adults acclimated to 32°C was significantly lower than the mass of adults acclimated to 28°C (*F*_(1)_ = 5.43, *P* = .04) Temperature, however, had no significant effect on length at any stage (all *P* > .05).

**Table 2:** Mass (g) and body length (mm) of individuals used for respirometry experiments.

	Mass	Length	T/I	Temperature
	(g)	(mm)		(°C)
Larvae	–	8.40 ± 0.29	5/5	28
	–	8.00 ± 0.17	5/5	32
Juveniles	–	14.32 ± 1.08	3/5	28
	–	13.45 ± 0.09	4/5	32
Adults	0.37 ± 0.01	36.84 ± 0.70	5/5	28
	0.32 ± 0.02	36.04 ± 0.36	5/5	32

T, number of replicate tanks, or shoals. I, number of individuals per shoal. Individual values were averaged for each shoal. Temperature indicates the target treatment temperature in °C. Values are given as mean ± SD.

### Oxygen consumption rates

**Fig. 3:**
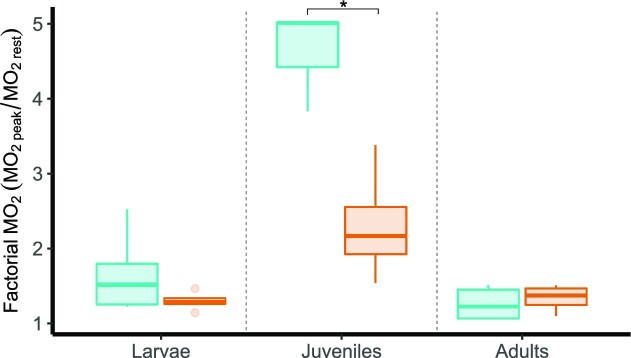
Factorial metabolic rates (*MO*_2_) were calculated as the ratio between the *MO*_2_ peak during recovery and the resting routine *MO*_2_ across ontogeny (larvae, juveniles, and adults) at 28°C and 32°C. Factorial *MO*_2_ is significantly lower at 32°C than 28°C in juveniles as the result of thermal sensitive resting routine metabolic rates in juveniles (*Q*_10_ = 9.98). Asterisks indicate significant differences between temperatures at each stage (α = .05).

Resting routine oxygen consumption rates (*MO*_2*rest*_) were significantly greater at the higher temperature in adults and larvae, but there was no significant effect of temperature on metabolic rates of juveniles (One-Way ANOVA, adults: *F*_(1, 7)_ = 7.38, *P* = .03; juveniles: *F*_(1, 5)_ = 6.06, *P* = .05; and larvae: *F*_(1, 8)_ = 14.82, *P* = .005, see Supplementary Table 1). The peak of oxygen consumption during recovery from exercise (*MO*_2*peak*_) was significantly greater at 32°C only in adults (tests for each stage separately because the *MO*_2_ are not mass-adjusted; One-Way ANOVA, adults: *F*_(1, 7)_ = 5.64, *P* = 0.049; juveniles: *F*_(1, 5)_ = 0.75, *P* = .4; and larvae: *F*_(1, 8)_ = 0.003, *P* = .9). Overall the factorial metabolic rate, factorial *MO*_2_ (*MO*_2*peak*_/*MO*_2*rest*_) is affected by stage and temperature (Two-way ANOVA, *F*_(5, 20)_ = 26.84). Factorial *MO*_2_ showed a significant interaction between temperature and stage (*P* = .0001), reflecting the higher thermal sensitivity of the juvenile *MO*_2_ (Fig. 3). In fact, factorial *MO*_2_ is significantly lower at 32°C in juveniles (*P* = .0001).

Thermal sensitivity (*Q*_10_) of resting routine *MO*_2_ showed a typical doubling of rates at 32°C from 28°C (larval *Q*_10_ = 1.96; adult *Q*_10_ = 2.19), with the exception of juvenile *Q*_10_ that displayed high thermal sensitivity (*Q*_10_ = 9.98). Mean peak *MO*_2_ during recovery showed a typical sensitivity to temperature in adults (*Q*_10_ = 2.53), but low sensitivity in larvae and juveniles (*Q*_10_ = 1.03 and 1.37, respectively).

### Shoal swimming kinematics

We analyzed video sequences at four to six speeds and two temperatures for larvae, juveniles, and adults (shoals of five fish each, *N* = 234 videos analyzed). For each speed and at each temperature, we analyzed 1–2 videos and averaged the results per shoal and speed.

**Fig. 4:**
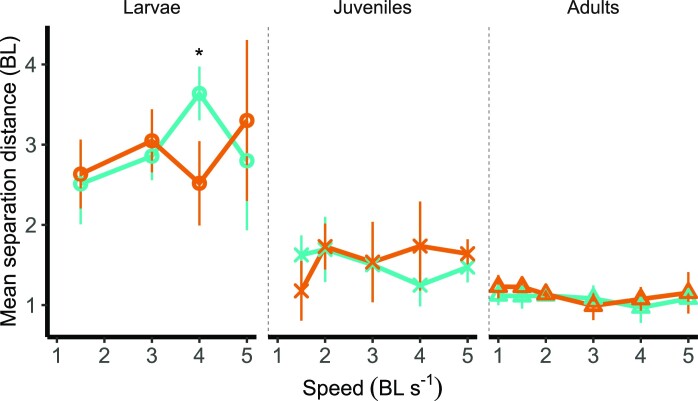
Mean separation distance (in body length, BL) between zebrafish *Danio rerio* in a shoal, decreases from larvae to juveniles and adults, across acclimation temperatures of 28°C (teal) and 32°C (orange). Asterisks indicate significant differences between temperatures at each stage (α = .05). Values displayed are means ±SD .

For each speed, we analyzed the impact of temperature and ontogenetic stage on swimming kinematics, mean separation distance, and switch rate. Mean separation distance was significantly different across stages from 1.5 to 5 BL s^−1^ (See Supplementary Table 2) and decreased in the shoal from larval to juvenile to adult stage (Fig. 4). We also report a significant interaction between ontogenetic stage and temperature on mean separation distance between specimens swimming at 4 BL s^−1^ (*P* = .0003, see Supplementary Table 2).

**Fig. 5:**
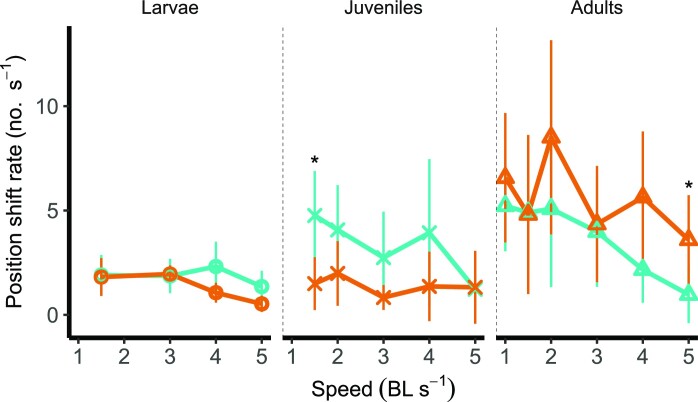
Number of position changes within a shoal in *Danio rerio* increases from larvae to adults. Asterisks indicate significant differences between the two treatment temperatures (teal: 28°C, orange: 32°C) for each speed (α = .05). Values displayed are means ± SD.

The rate of position shifts significantly differed between stages from 1.5 to 5 BL s^−1^ (See Supplementary Table 2) and doubled over ontogenetic time from larvae to adults (Fig. 5). We found an interaction between temperature and ontogenetic stage at 4 BL s^−1^, where larvae swam closer to each other at control temperature (*F*_(5, 23)_ = 2.93, *P* = .039).

**Fig. 6:**
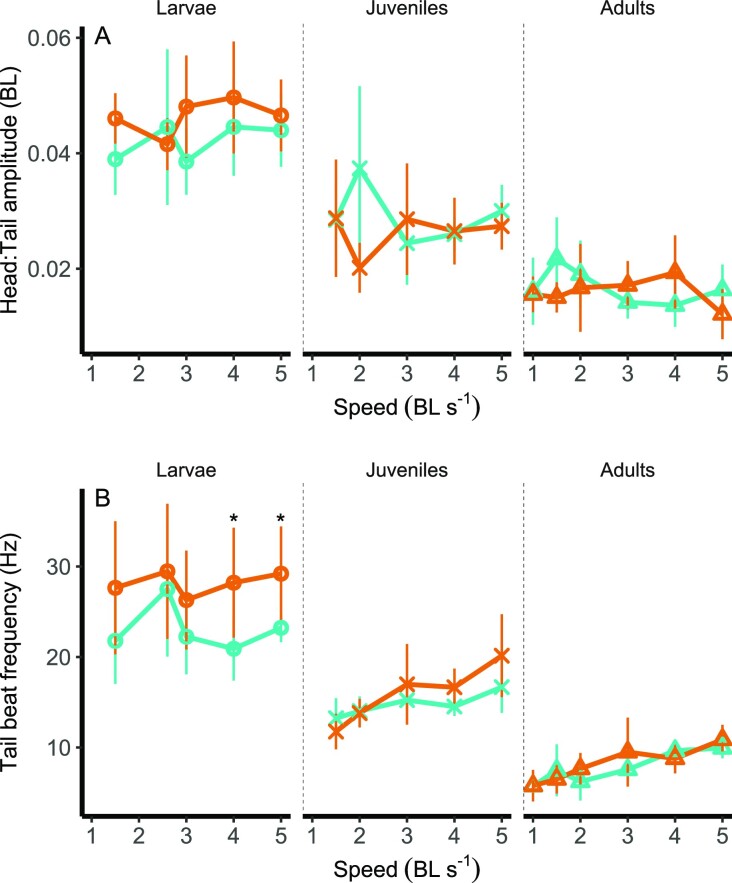
Ontogenetic shifts of head to tail amplitude (BL, or body length, A) and TBF (Hz, B) in shoaling zebrafish *Danio rerio*. Asterisks indicate significant differences between the two treatment temperatures, for each speed. Teal shows control acclimation temperature (28°C, teal) and orange represents higher acclimation temperature (32°C, orange). Asterisks indicate significant differences between temperatures at each stage (α = .05). Values displayed are means ± SD.

Major swimming kinematic features show a different response depending on the ontogenetic stage, temperature, and flow speeds. Head:Tail amplitude significantly decreased over ontogeny, at all speeds tested (see Supplementary Table 2 for significance values at different speeds) (Fig. 6 A). For instance, at 1.5 BL s^−1^, Head:Tail decreased from 0.04 BL in larvae to 0.03 BL in juveniles and 0.02 in adults (Stage *F*_(2)_ = 34.15, *P* < .001). Head:Tail amplitude was significantly higher at 28°C, at 2 BL s^−1^ (Temperature *F*_(1)_ = 5.46, *P* = .03) and lower at 32°C, at 3 BL s^−1^ (*F*_(1)_ = 4.59, *P* = .04).

Similarly, TBF significantly decreased over ontogeny, at all speeds (see Supplementary Table 2 for significance values at different speeds) (Fig. 6 B). TBF was also significantly higher at 32°C than 28°C at 4 BL s^−1^ (Temperature *F*_(1)_ = 5.538, *P* = .02) and 5 BL s^−1^ (Temperature *F*_(1)_ = 8.036, 1, *P* = .01). We also found an interaction between ontogenetic stage and temperature at 4 BL s^−1^, with TBF increasing with temperature in larvae but not in juveniles and adults (Interaction *F*_(2)_ = 3.99, *P* = .03).

**Fig. 7:**
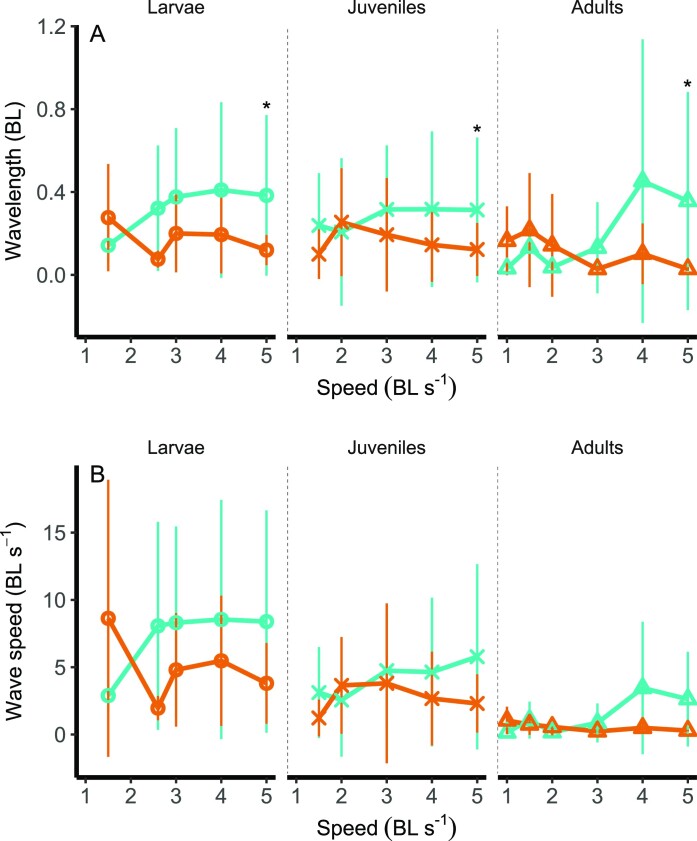
Ontogenetic shifts of wave speed (A) and wavelength (B) in *Danio rerio*. Wavelength is significantly lower at 32°C than 28°C in larvae. Teal, acclimatized to 28°C; orange, acclimatized to 32°C. BL, body length. Asterisks indicate significant differences between temperatures at each stage (α = .05). Values displayed are means ± SD.

Wave speed (Fig. 7 A) was significantly higher in juveniles when compared to adults swimming at 2 BL s^−1^ (*F*_(1)_ = 4.7, *P* = .04) and decreased from larvae (mean = 6.5 BL s^−1^) to juveniles (mean = 4.3 BL s^−1^) and adults (mean = 0.6 BL s^−1^) at 3 BL s^−1^ (*F*_(2)_ = 4.2, *P* = .03, see Supplementary Table 2 for significance values at different speeds).

Wavelength (Fig. 7 B) was significantly higher in fish acclimatized to 28°C than 32°C, when swimming at 5 BL s^−1^ (Temperature *F*_(1)_ = 4.82, *P* = .04, see Supplementary Table 2 for significance values at different speeds).

Maximum curvature of the body was significantly lower at 28°C when compared to 32°C when fish swam at 5 BL s^−1^ (*F*_(1)_ = 4.82, *P* = .039, see Supplementary Table 2 for significance values at different speeds).

## Discussion

Assessing the role of ontogeny and the impact of warming on shoaling behavior is crucial to forecast the likely responses and vulnerabilities of fishes under climate change across their life. In this work, a small freshwater forage fish shifts its shoaling behavior across ontogeny and under warming. During the larval phase, zebrafish generate cyclical body undulations and, as they grow, they rely on burst and coast swimming to reduce the costs of locomotion, as seen in juveniles and adults (Müller and van Leeuwen 2004; Burgerhout et al. 2013). Shifts in collective behavior, such as changes in mean separation distance (shoal cohesion, Fig. 4), rates in changes in position (formation destabilization, Fig. 5), and efficiency of motion could all negatively impact swimming performance. Warming and life stages showed complex interactive effects at different speeds. For instance, under warming mean separation distance were lower in larvae at 4 BL s^−1^ than at control temperature, while position shift rates were higher only in adults at 5 BL s^−1^. Position shifts were lower in juveniles at low speeds and warming. At the the higher temperature TBF of larvae increased at high speeds, which was already significantly higher than TBF of juveniles and adults across speeds. It is plausible that a combination of higher mean separation distance coupled with the inability of shifting position to take advantage of areas of reduced flow at high speeds might have contributed to high TBF, and hence low efficiency, at the high temperature in larval zebrafish. The effect of warming on shoaling behavior could be caused by complex interactions at different stages between energetics and biomechanical parameters. This results in low efficiency in larval shoaling, especially under warming conditions.

Furthermore, in zebrafish, mean separation distance (Fig. 4) decreases from larvae to adults, suggesting that cohesion increases across the lifetime of the fish. This stands in contrast with other work that suggested that the development of shoaling behavior might be “completed” during the larval phase (Masuda et al. 2003). Different species may complete shoaling development at different rates. As shoaling is crucial to increase survival rates during early life stages, it is important to understand the mechanisms underlying the variation in shoaling timing in different groups of fishes. Cohesion is the most important parameter when considering the efficacy of collective motion from an energetic standpoint. Individual fish can reduce their own energy expenditure in a formation by holding positions behind other swimming fish to take advantage of vortices within the shoal (Weihs 1973), however, it is the shortening of the distance between individuals (higher cohesiveness) that increases the efficiency of the whole group (Di Santo 2022). In our study, individuals of all ontogenetic stages were capable of shoaling, which naturally occurs post-flexion in larval zebrafish (Miller and Gerlai 2007; Buske and Gerlai 2011). However, zebrafish improve their shoaling behavior over ontogeny, regardless of temperature, as evidenced by the three-fold shortening of distance between individuals from the larval to the adult stage.

The improvement in shoaling behavior is reflected in the much more efficient recovery from exercise in adult zebrafish when compared to juveniles (Fig. 3). Such improvement might be the result of a smaller sensitivity to temperature observed in adults, but also of the ability of adult zebrafish to select more advantageous positions within the formation (Fig. 5) and the shortening of the distances between individuals (Fig. 4). In the wild, zebrafish exhibit a more cohesive aggregation in high-flow and complex environments which may help them save energy during locomotion. Similarly, guppies (*Poecilia reticulata*) and chub (*Squalius cephalus*) only form shoals when they are challenged by high flow (Allouche and Gaudin 2001; Hockley et al. 2014). In our study, there was a reduction in rates of position shifts within the formation in juveniles and adults as speed increased (Fig. 5). However, instability of the group increased in adults kept under warming conditions. Warming increases metabolic rates during and after swimming exercises and prolongs the time to recovery (Zeng et al. 2010; Pang et al. 2015; Di Santo 2016). Hence, it is possible that the dynamic shifts in the shoal may be the consequence of individuals trying to compete for energetically advantageous positions (Herskin and Steffensen 1998), for instance behind other fish (Di Santo 2022). In other words, it is possible that by being more experienced shoalers, adults may decide to break the formation to find a better position, causing the shoal to become more volatile. Therefore, individuals would need to balance propulsive efficiency within stable formations with the necessity to compensate for elevated metabolic rates at higher speeds. Juvenile zebrafish, on the other hand, reduced the frequency of position changes at higher speeds or temperatures, while larvae only responded to changes in water velocity by maintaining more stable positioning. These results suggest that warming and flow impose different and interacting effects on zebrafish depending on their life stage.

Major kinematic features showed large variations in temperature and ontogenetic stages in zebrafish. TBF decreased ontogenetically across temperatures (Fig. 6 B). Because larvae maintain much looser shoals, they may experience greater negative effects of temperature on their movement when compared to juveniles that swim closer to each other (Fig. 4).

From this study, it is clear that warming exerts a different effect on shoaling behavior of zebrafish across ontogeny. More work is now needed to clarify how changes in shoaling performance might affect the daily and seasonal movements of forage fishes. Furthermore, fishes are already coping with an array of anthropogenic stressors that are altering the chemical and physical environment in which they live. The effects of warming tend to be stronger during earlier life stages and this may represent a potential bottleneck for the survival and recruitment of shoaling species. In this study, parental and developmental effects were not tested, but these may reduce the negative effect of warming on early life stages and should be further explored. More studies may investigate the long-term consequences of warming and other climate-related stressors on shoaling behavior, kinematics, and resilience.

## Conclusions

Shoaling behavior improves in zebrafish as they transit from larvae to juveniles and to adults. Our study provides evidence for enhanced collective behavior, primarily manifested by a decrease in mean separation distance and positional shifts, particularly at higher speeds. These findings suggest that as zebrafish grow, they are better able to exploit the shoal formation to enhance locomotor efficiency. Temperature exerts a significant effect on energetics, behavior, and kinematics of shoaling behavior in zebrafish. While thermal sensitivity *Q*_10_ in larvae and adults followed a typical doubling in metabolic rates, juvenile resting metabolic rates were exceptionally sensitive to warming. To date, the effect of temperature on fish swimming biomechanics has only been explored in a few systems (Di Santo 2022). However, studies have now shown that routine, escape, and sustained locomotor behaviors can all be affected by warming (Brett 1971; Bartolini et al. 2015; Andreassen et al. 2022). Nonetheless, further research is necessary to fully comprehend the extent of these effects and their implications. To advance our understanding of collective locomotion, future investigations should encompass a comprehensive approach integrating energetics, biomechanics, behavior, and computational analyses. This integrative approach will enable a more thorough examination of the alterations and resilience of shoaling behavior in response to temperature changes.

## Supplementary Material

icad043_Supplemental_File

## Data Availability

Data supporting this work are available online at https://github.com/fberio/zebrafishShoaling.

## Notes

From the symposium “Pathways to adulthood: environmental, developmental, and evolutionary influences on the ontogeny of form and function” presented at the annual meeting of the Society for Integrative and Comparative Biology virtual annual meeting, January 16–March 31, 2023.
