# Supplementary material for: Ontogenetic Plasticity in Shoaling Behavior in a Forage Fish under Warming
Source: Integr Comp Biol. 2023 May 27;63(3):730–41. doi: 10.1093/icb/icad043 (PMC10503471; doi:10.1093/icb/icad043)
Supplement: icad043_Supplemental_File [file icad043_supplemental_file.pdf]

Variable	factor(s)	stages	F	p-val	df
Factorial MO_2	temperature	all	22.2663	0.0001	1
	stage	all	51.5869	< 0.0001	2
	stage, temperature	all	14.3557	0.0001	2
	model	all	26.8409	< 0.0001	5, 20
MO_{2rest} average	temperature (model)	adults	7.3834	0.0299	1, 7
	temperature (model)	juveniles	6.0633	0.0571	1, 5
	temperature (model)	larvae	14.8250	0.0049	1, 8
max MO_{2rec}	temperature (model)	adults	5.6498	0.0491	1, 7
	temperature (model)	juveniles	0.7516	0.4256	1, 5
	temperature (model)	larvae	0.0036	0.9535	1, 8

Table 1: Supplementary table: Detailed results of statistical analyses (One-Way and Two-Way ANOVAs) on oxygen consumption rates.

Variable	speedBL	factor(s)	stages	F	p-val	df
Head to tail amplitude	1	temperature (model)	adults	0.034	0.858	1
	1.5	stage	all	34.159	$9.53e^{-8}$	2
	1.5	temperature	all	0.009	0.9272	1
	1.5	stage, temperature	all	2.742	0.0846	2
	1.5	model	all	14.7622	< 0.0001	5, 24
	2	stage	adults, juveniles	6.813	0.0197	1
	2	temperature	adults, juveniles	5.467	0.0336	1
	2	stage, temperature	adults, juveniles	3.182	0.0947	1
	2	model	adults, juveniles	5.4334	0.0099	3, 15
	2.6	temperature (model)	larvae	0.259	0.623	1
	3	stage	all	39.867	$4.84e^{-8}$	2
	3	temperature	all	4.595	0.0434	1
	3	stage, temperature	all	0.632	0.5409	2
	3	model	all	17.4817	< 0.0001	5, 22
	4	stage	all	52.484	$4.23e^{-9}$	2
	4	temperature	all	2.211	0.151	1
	4	stage, temperature	all	0.400	0.675	2
	4	model	all	22.0907	< 0.0001	5, 22
	5	stage	all	86.564	$3.74e^{-11}$	2
	5	temperature	all	0.545	0.468	1
	5	stage, temperature	all	1.145	0.337	2
	5	model	all	34.8514	< 0.0001	5, 22
	1	temperature (model)	adults	0.002	0.968	1, 8
	1.5	stage	all	51.288	$2.16e^{-9}$	2
	1.5	temperature	all	0.601	0.4458	1
	1.5	stage, temperature	all	2.621	0.0934	2
	1.5	model	all	21.6838	< 0.0001	5, 24
	2	stage	adults, juveniles	72.114	$4.1e^{-7}$	1

TBF

	2	temperature	adults, juveniles	0.528	0.479	1
	2	stage, temperature	adults, juveniles	1.005	0.332	1
	2	model	adults, juveniles	24.8251	< 0.0001	3, 15
	2.6	temperature (model)	larvae	0.188	0.675	1
	3	stage	all	42.464	$2.8e^{-8}$	2
	3	temperature	all	3.331	0.0816	1
	3	stage, temperature	all	0.286	0.7541	2
	3	model	all	18.1439	< 0.0001	5, 22
	4	stage	all	55.377	$2.59e^{-9}$	2
	4	temperature	all	5.538	0.028	1
	4	stage, temperature	all	3.998	0.033	2
	4	model	all	25.0530	< 0.0001	5, 22
	5	stage	all	56.930	$2.01e^{-9}$	2
	5	temperature	all	8.036	0.00964	1
	5	stage, temperature	all	1.448	0.25659	2
	5	model	all	25.3719	< 0.0001	5, 22
	1	temperature (model)	adults	3.834	0.0859	1
	1.5	stage	all	3.095	0.0637	2
	1.5	temperature	all	0.504	0.4844	1
	1.5	stage, temperature	all	1.979	0.1601	2
	1.5	model	all	2.1305	0.0963	5, 24
	2	stage	adults, juveniles	4.718	0.0463	1
	2	temperature	adults, juveniles	0.345	0.5655	1
	2	stage, temperature	adults, juveniles	0.076	0.7864	1
	2	model	all	1.6485	0.2205	3, 15
Wave-speed	2.6	temperature (model)	larvae	3.771	0.0841	1
	3	stage	all	4.060	0.0316	2
	3	temperature	all	0.936	0.3437	1
	3	stage, temperature	all	0.282	0.7566	2
	3	model	all	1.9367	0.1288	5, 22
	4	stage	all	2.071	0.150	2
	4	temperature	all	1.629	0.215	1
	4	stage, temperature	all	0.028	0.973	2
	4	model	all	1.1210	0.3783	5, 22
	5	stage	all	1.984	0.1614	2
	5	temperature	all	3.256	0.0849	1
	5	stage, temperature	all	0.117	0.8904	2
	5	model	all	1.4743	0.2383	5, 22
	1	temperature (model)	adults	3.19	0.112	1
	1.5	stage	all	0.114	0.892	2
	1.5	temperature	all	0.124	0.728	1
	1.5	stage, temperature	all	1.235	0.309	2
	1.5	model	all	0.5644	0.7262	5, 24
	2	stage	adults, juveniles	1.465	0.245	1
	2	temperature	adults, juveniles	0.432	0.521	1

Wave-
length

	2	stage, temperature	adults, juveniles	0.063	0.806	1
	2	model	all	0.6452	0.5979	3, 15
	2.6	temperature (model)	larvae	3.947	0.0782	1
	3	stage	all	1.833	0.184	2
	3	temperature	all	1.974	0.174	1
	3	stage, temperature	all	0.055	0.946	2
	3	model	all	1.1433	0.3676	5, 22
	4	stage	all	0.077	0.926	2
	4	temperature	all	2.663	0.117	1
	4	stage, temperature	all	0.124	0.884	2
	4	model	all	0.6059	0.6961	5, 22
	5	stage	all	0.081	0.9222	2
	5	temperature	all	4.580	0.0437	1
	5	stage, temperature	all	0.102	0.9032	2
	5	model	all	0.9762	0.4540	5, 22
Maxcurv	1	temperature (model)	adults	1.372	0.275	1, 8
	1.5	stage	all	1.410	0.264	2
	1.5	temperature	all	0.071	0.792	1
	1.5	stage, temperature	all	1.515	0.240	2
	1.5	model	all	1.1843	0.3461	5, 24
	2	stage	adults, juveniles	0.596	0.452	1
	2	temperature	adults, juveniles	3.033	0.102	1
	2	stage, temperature	adults, juveniles	1.128	0.305	1
	2	model	adults, juveniles	1.6080	0.2294	3, 15
	2.6	temperature (model)	larvae	1.428	0.263	1
	3	stage	all	1.629	0.219	2
	3	temperature	all	1.545	0.227	1
	3	stage, temperature	all	0.202	0.818	2
	3	model	all	1.0613	0.4081	5, 22
	4	stage	all	0.788	0.467	2
	4	temperature	all	1.118	0.302	1
	4	stage, temperature	all	0.265	0.770	2
	4	model	all	0.6776	0.6449	5, 22
	5	stage	all	1.714	0.2033	2
	5	temperature	all	4.823	0.0389	1
	5	stage, temperature	all	0.410	0.6686	2
	5	model	all	1.9284	0.1302	5, 22
Mean separation distance	1	temperature (model)	adults	1.686	0.23	1, 8
	1.5	stage	all	49.667	$4.5e^{-9}$	2
	1.5	temperature	all	0.308	0.585	1
	1.5	stage, temperature	all	2.202	0.133	2
	1.5	model	all	21.0708	< 0.0001	5, 23
	2	stage	adults, juveniles	27.910	$9.2e^{-5}$	1
	2	temperature	adults, juveniles	0.053	0.82	1
	2	stage, temperature	adults, juveniles	0.008	0.93	1

	2	model	adults, juveniles	9.4475	0.0009	3, 15
	3	stage	all	80.431	$4.15e^{-11}$	2
	3	temperature	all	0.119	0.733	1
	3	stage, temperature	all	0.405	0.672	2
	3	model	all	32.3601	< 0.0001	5, 23
	4	stage	all	82.017	$3.41e^{-11}$	2
	4	temperature	all	1.581	0.221295	1
	4	stage, temperature	all	12.069	$2.61e^{-4}$	2
	4	model	all	37.8138	< 0.0001	5, 23
	5	stage	all	31.041	$2.93e^{-7}$	2
	5	temperature	all	1.339	0.259	1
	5	stage, temperature	all	0.383	0.686	2
	5	model	all	12.8175	< 0.0001	5, 23
Switch rate	1	temperature (model)	adults	0.6317	0.4497	1,8
	1.5	stage	all	3.739	0.0393	2
	1.5	temperature	all	0.750	0.3953	1
	1.5	stage, temperature	all	1.182	0.3245	2
	1.5	model	all	2.6793	0.0475	5, 23
	2	stage	adults, juveniles	7.826	0.0135	1
	2	temperature	adults, juveniles	0.256	0.6201	1
	2	stage, temperature	adults, juveniles	1.704	0.2114	1
	2	model	adults, juveniles	3.0337	0.0620	3, 15
	3	stage	all	5.651	0.0101	2
	3	temperature	all	0.119	0.7329	1
	3	stage, temperature	all	0.732	0.4919	2
	3	model	all	2.3341	0.0747	5, 23
	4	stage	all	3.570	0.0447	2
	4	temperature	all	0.008	0.9313	1
	4	stage, temperature	all	3.760	0.0387	2
	4	model	all	2.9392	0.0340	5, 23
	5	stage	all	2.434	0.110	2
	5	temperature	all	0.414	0.526	1
	5	stage, temperature	all	2.046	0.152	2
	5	model	all	3.2428	0.0232	5, 23

Table 2: Supplementary table: Detailed results of statistical analyses (One-Way and Two-Way ANOVAs) on kinematics, mean separation distance, and number of switch rates. TBF, tail beat frequency; speedBL, speed in body length.
